# Timing of First Antenatal Care Attendance and Associated Factors among Pregnant Women in Arba Minch Town and Arba Minch District, Gamo Gofa Zone, South Ethiopia

**DOI:** 10.1155/2015/971506

**Published:** 2015-10-12

**Authors:** Feleke Gebremeskel, Yohannes Dibaba, Bitiya Admassu

**Affiliations:** ^1^College of Medicine and Health Sciences, Arba Minch University, P.O. Box 021, Arba Minch, Ethiopia; ^2^College of Health Sciences, Jimma University, P.O. Box 378, Jimma, Ethiopia

## Abstract

*Objective*. To assess the timing of first antenatal care attendance and associated factors among pregnant women in Arba Minch Town and Arba Minch District, south Ethiopia. *Method*. Facility based cross-sectional study employing both quantitative and qualitative methods was conducted from February to March, 2014, in Arba Minch Town and Arba Minch District. Data were collected from 409 pregnant women attending antenatal care clinics in nine public health facilities using systematic random sampling. Analysis was done using SPSS version 20. Descriptive statistics and binary and multiple logistic regression analysis were done. *Results*. The mean (SD±) age of the respondents was 26 ± 5.5 years. The mean gestational age at first antenatal care attendance was 5 ± 1.5 months. This study indicated that pregnant women with low monthly income (AOR = 4.9, CI: 1.71, 14.08), women who did not receive advise on when to start ANC (AOR = 3, CI: 1.48, 6.24), women with household food insecurity (AOR = 4.66, CI: 1.007, 21.59) and women with unplanned pregnancy (AOR = 4.49, CI: 2.16, 9.35) had higher odds of late antenatal care attendance compared with their counterparts. *Conclusions*. The study showed that majority of the pregnant women attended late for first antenatal care. Hence, providing health education on the timing of antenatal care is important.

## 1. Introduction

Maternal mortality is a major health problem in sub-Saharan Africa. According to the recent estimate by the maternal mortality estimation interagency group, the number of maternal deaths worldwide has declined to 289,000 in 2013, representing 45% decline from the 1990 level [1]. About 62% of these deaths occurred in sub-Saharan Africa where the maternal mortality ratio still stands at 510 deaths per 100,000 live births. Moreover, majority of the countries with the highest maternal mortality are in sub-Saharan Africa, including Ethiopia [1, 2]. In 2011, the maternal mortality ratio in Ethiopia was estimated at 676 deaths per 100,000 live births [3]. Most of these deaths occur unpredictably during labor, delivery, and the immediate postpartum period. Although efforts have been made to achieve the Millennium Development Goal (MDG5) on maternal health to reduce the maternal mortality ratio (MMR) by three-quarters by 2015, this goal has not been achieved yet. This is mainly because of the low utilization of maternal health services. Quality reproductive and maternal health care throughout pregnancy, childbirth, and the postpartum period can effectively ensure positive health outcomes for women and their children [1, 4, 5].

Pregnancy is one of the most important periods in the life of a woman, a family, and a society. Antenatal care (ANC) is special care for women during pregnancy through the public health services. The goal of ANC is to prevent health problems in both the fetus and mother and to ensure that each newborn child has a good start [5–7]. World Health Organization (WHO) recommended that pregnant women in developing countries should seek ANC within the first three months of pregnancy. For programme areas in particular, namely, tuberculosis (TB), nutrition, immunization against tetanus, and prophylactic treatment of malaria and human immune virus/acquired immune deficiency syndrome (HIV/AIDS) and other sexually transmitted infections (STIs), the antenatal period represents an important opportunity, yet it currently appears to be underexploited [7, 8].

Moreover, planning for a safe delivery is an integral part of ANC. Early antenatal care attendance during the first three months of gestation plays a major role in detecting and treating some complications of pregnancy and forms a good basis for appropriate management during delivery and after childbirth. Failure to attend antenatal care early results in the potential for complications during pregnancy, delivery, and puerperium [6, 9]. However, existing evidence from developing countries including Ethiopia indicates that few women seek antenatal care at early stage of their pregnancy [3, 10, 11]. According to the 2011 Ethiopia Demographic Health Survey, 19% of women with a live birth in the five years before the survey made four or more ANC visits during their recent pregnancy, while only 11% made their first ANC visit before the fourth month of pregnancy. The median duration of pregnancy at the first visit was 5.2 months, and urban women made their first ANC visit earlier (4.4 months) than rural women (5.5 months) [3].

Studies have identified several factors that influence the utilization of antenatal care in developing countries, although there are few studies regarding factors affecting the timing of first ANC attendance. These factors include, among others, maternal education, husband's education, availability of health service, cost, household income, women's employment, media exposure, and having a history of obstetric complications [12–15]. Although there is limited evidence, late booking of antenatal care has been associated with young age, premarital status, unwanted pregnancies, high parity, lack of formal education, low socioeconomic status, and ethnicity [16–18]. Unintended pregnancy also had an influence on antenatal care use; this may lead mothers to attend antenatal care late or to not attend at all [10, 19]. The quality of antenatal care might have an influence on utilization of antenatal care, leading to infrequent or late first visits to antenatal care [18, 20–22].

Even in the urban areas of Ethiopia, where the health services are physically accessible and available and also maternal health services in public facilities are provided for free, early ANC attendance is still low [18, 23]. These studies identified several barriers to early ANC attendance. A study carried out in Addis Ababa (Ethiopia) revealed that respondents who received advice on recommended time were more likely to start ANC timely than those who had no information or advise about ANC [18]. Another study in Bahir Dar, Ethiopia, showed that the likelihood of delivering at home was greater among mothers who started attending ANC after 24 weeks of gestation [23]. There were no studies on the magnitude of early ANC initiation and associated factors in southern Ethiopia and in the study area in particular. Late initiation of ANC may lead to undetected or late detection of maternal health problems and subsequently unmanaged complication among pregnant women and thus contributes to maternal mortality. Thus, the aim of this study was to examine factors associated with delay in first ANC attendance.

## 2. Methods

### 2.1. Study Setting

The study was conducted in Arba Minch Town and Arba Minch District public health facilities, Gamo Gofa Zone, in Southern Ethiopia. Arba Minch is a town located 505 Km away from Addis Ababa and 275 km south west of Hawassa, capital city of the southern regional state. Facility based cross-sectional study design was employed using quantitative and qualitative methods. The study was carried out among selected pregnant women attending ANC service in public health facilities from February to march, 2014. Pregnant women who attended first ANC visits in other health facilities who were seriously ill and unable to respond were excluded.

### 2.2. Sample Size and Sampling Technique

Sample size for the study was calculated using single population proportion formula with the following assumptions. Based on findings from a previous study in Bahir Dar (Ethiopia) the proportion of women who start ANC after the fourth month was found to be 59% [24]. By assuming a margin of error of 5%, *Z*_*α*/2_ = value for 95% CI (1.96), proportion = 59%, and nonresponse rate of 10%, a sample size of 409 pregnant women obtained. Then, stratified systematic sampling was employed to select the study participants from each health facility with total of nine public health facilities. The sampling interval (*k* = *N*/*n* = 2) was used in nine public health facilities to select study subjects. The first client was selected by lottery method among the first two ANC service users. Purposive sampling method was used to select ANC providers who have more experience in ANC unit in health facility and health extension workers who are working in the district for qualitative data.

The outcome variable of this study was late first ANC attendance (starting ANC after 16 weeks of gestation), while the independent variables include sociodemographic and economic factors such as age, educational status, occupation, marital status, religion, ethnicity, place of residence, monthly income, and family size; health service related factors such as client satisfaction with ANC services, availability of Health Care Providers, distance of health facility, payment for ANC, waiting time, and presence of resources; maternal factors such as previous use of service, culture, belief, knowledge towards ANC, means of awareness of pregnancy, perceptions on use of early seeking of ANC, advise from significant others, and timing of pregnancy recognition; obstetric related factors such as acceptance of pregnancy by significant others, birth order, type of pregnancy, gravidity, parity, and bad obstetric history (abortion and still birth); and household food insecurity status was considered as independent variable.

### 2.3. Data Collection

The quantitative data were collected using a structured interview administered questionnaire. The questionnaire was translated to local language and pretested before field work. Nine female nurses, trained for two days, conducted the exit interview with pregnant women. The interviews were carried out in a private and calm environment so as to ensure confidentiality. For participants who did not recall their gestational age, their medical records were reviewed on the same day to collect further information on gestational age during their first ANC visit after having consent. Household food security scale was used to measure food security status [25]. For qualitative data open ended interview guide was used for the in-depth interview with pregnant women, ANC clinic workers, and health extension workers. In-depth interview with six pregnant women, four antenatal care providers, and four health extension workers was also carried out in the study area until the information was saturated.

### 2.4. Data Analysis

Epidata3.1 and SPSS version 20 were used for data entry and analysis, respectively. Frequency distributions of variables of interest were computed to describe the characteristics of the respondents. Binary logistic regression analysis was used to identify variables that are significant with the outcome variable at *p* value ≤ 0.25 and those variables were considered for the final model. Finally, multiple logistic analyses were carried out to identify the predictors of late ANC booking. Backward stepwise regression method was used to test the model's fitness. Those variables with *p* value ≤ 0.05 with adjusted odds ratio and 95% confidence interval were considered as statistically significant. The in-depth interviews were audio taped, transcribed, translated, and coded into larger themes and categories, analyzed thematically by open code version 3.4 and triangulated with quantitative data.

Ethical clearance was obtained from the Ethical Review Committee of Jimma University. Permission letter and informed verbal consent were obtained from zonal health department. All informants were ensured anonymity and confidentiality, and they gave their informed consent after appropriate explanation of the study objectives and content.

## 3. Results

Four hundred nine pregnant women were interviewed in this study. Out of those 59 (14.4%) were in the age range between 15 and 19 years, 110 (26.9%) were in the age range between 20 and 24 years, 127 (31.1%) were in the age range between 25 and 29 years, and 81 (18.9%) were in the age range between 30 and 34 years. The mean (±SD) age of pregnant women was 26 (±5.5) years. The ethnic composition of study participants shows that Gamo (76%) are the dominant group followed by Welaita (10.8). Around 47.2% and 46.9% of participants were followers of Orthodox and Protestant religions, respectively. Majority of pregnant women 384 (93.9%) were married. Regarding educational status, 117 (28.6%) and 134 (32.8%) were illiterate and had attended primary school, respectively. Majority of the respondents (57.2%) were rural residents. Nearly half of pregnant women (45%) had monthly income of above 1425 ETB (Table 1).

Thirty-seven percent (37.1%) of respondents were gravida one, while the rest (62.9%) were two and above. One hundred seventy-two (42%) of respondents were nulliparous and the rest 237 (58%) had one or more children. Sixty-one (15%) of respondents had history of at least one abortion.

### 3.1. Timing of First ANC Attendance

Information on timing of first ANC was collected from the women's recall or client's card if they could not remember the exact dates. Accordingly, the proportion of respondents who made their first ANC visit within recommended time (before 16 weeks of gestation) was 17.4%.

Timing of first ANC attendance ranges from 1st month to 9th month during pregnancy. Around thirty percent (29.9) of the pregnant women attended first ANC visits at four months of gestation. The mean duration (±SD) of first ANC attendance was 5 months (±1.5). Three hundred seventeen (75.5%) of pregnant women received advice about ANC use from any one before their first ANC visit. Majority of pregnant women (58%) received advice from community health workers, followed by husband (24%). Two hundred eighty-two (89%) pregnant women reported that they were advised when to start first ANC visit. Among those women who were advised, 47% were advised before 16 weeks of gestation. In this study, 71.1% of pregnant women were aware of danger signs during pregnancy. Among those women who told us the danger signs, 53.6% were aware of all types of danger signs of pregnancy.

### 3.2. Past History of ANC Service Utilization

Among two hundred fifty-seven pregnant women (62.8%) who had history of previous pregnancy in their life, 88% had attended ANC for the previous pregnancy, while the remaining 12% did not attend ANC. Regarding the birth order of the women who attended ANC previously, 62% was first pregnancy, whereas 39% was second pregnancy. Out of 227 pregnant women who had history of ANC use in previous pregnancy, 34.4% had their previous ANC visit before 16 weeks of gestation, while the remaining 65.6% had their first ANC visit at or after 16 weeks of gestation. Fifty-two (52%) of pregnant women were satisfied with ANC services in terms of staff approach, laboratory service, privacy, waiting time, and service.

### 3.3. Current Pregnancy and ANC Utilization

Three hundred fifty-nine (87.8%) pregnant women reported that they recognized their current pregnancy when they missed menses (monthly period) for one to three months. Two hundred sixty-nine (66%) pregnant women reported that their pregnancies were planned. Majority of pregnant women (87.3%) informed their husbands of their pregnancy first before ANC visits. In addition, pregnant women who came to ANC in this time were asked what made them come in the first trimester, second trimester, and third trimester. Among those who booked late, most of them (61%) became late because they perceived that it is the right time to start, while (22%), (7%), (5%), and (4.7%) came because of lack of time, unawareness of pregnancy, learning from previous experience, and unplanned pregnancy.

### 3.4. Factors Associated with Late First Antenatal Care Attendance

In this study, the factors that were associated with late ANC attendance were low monthly income, receiving advice on when to start ANC visits, household food insecurity, and unplanned pregnancy. The odds of pregnant women with low household monthly income to delay ANC booking were 5 times (AOR = 4.9, CI: 1.713, 14.076) higher compared to their counterparts with high monthly income. Pregnant women who received advice not on recommended time were 3 times (AOR = 3, CI: 1.476, 6.244) more likely to book late for their first ANC as compared to their counterparts who received advice on recommended time. Pregnant women with unplanned pregnancy were 4.5 times (AOR = 4.49, CI: 2.162, 9.353) more likely to book late for their first ANC as compared to their counterparts with planned pregnancy. Pregnant women who are living in food-insecure household were around 5 times (AOR = 4.66, CI: 1.007, 21.589) more likely to be late for first ANC attendance as compared to women with food-secure household (Table 2).

The qualitative study also indicated some reasons why women come late for ANC. One of the health providers stated “…even though most of them knew the right gestation age when to start but they could not go to health facility at early time of pregnancy without feeling of the baby moving in their abdomen and do not want to show their body for health professionals at first trimester of pregnancy by health care provider.” This might be due to lack of knowledge and perception on importance of early antenatal care visits.

## 4. Discussion

According to recommendation of World Health Organization (WHO), a pregnant woman needs to start antenatal care in the first trimester of pregnancy. However, a significant proportion of women from developing countries do not start ANC according to the recommendation. This study revealed that 82.6% of the pregnant women initiated antenatal care at or after four months of gestation. This finding is higher than that in the studies done in Addis Ababa (Ethiopia), Bahir Dar (Ethiopia), Gonder (Ethiopia), and Nigeria [18, 22, 26, 27]. This might be due to sociodemographic, economic, and cultural differences as evidenced by the fact that majority of pregnant women had no education and only attained primary school; more than half of women living in rural areas were housewives as compared to Addis Ababa and northern Ethiopian residents. This study also showed that the mean gestational age at first antenatal care booking is 5 months. This result is consistent with the Ethiopian Demographic and Health Survey report that showed median duration of pregnancy at the first antenatal care booking of 5.2 months [3]. Household income was one of the factors significantly associated with late antenatal care entry in this study. Pregnant women who had low household monthly income were 5 times more likely to book late for their first ANC booking as compared to their counterparts with high monthly income. This result is in line with the study done in Metekel Zone (Ethiopia), Holeta town (Ethiopia), and Uganda [15, 28, 29] Furthermore, several other studies showed that low monthly income is associated with increased odds of underutilizing antennal care services among pregnant mother. This could be because of the fact that better income might increase the ability to pay for health care services, transportation, and other indirect costs. As compared to the proportion of pregnant women with past antenatal care experience before current pregnancy, those pregnant women without past antenatal care experience started ANC later than pregnant women with past experience for first antenatal care. This finding was supported by the study done in Addis Ababa and Uganda [18, 29].

Previous experience of antenatal care utilization might improve the current pattern of antenatal care and timing of first antenatal care visits. However, the difference in this study was not statistically significant in final model. This could be due to the fact that the pregnant mother thought there is no need to come early for antenatal care if one has no problem with the pregnancy. Majority of respondents reported that they received advice on when to start visits for antenatal care before being started by community health workers. The study showed that pregnant women who did not receive advice on recommended time were 3 times more likely to book late for their first ANC booking as compared to their counterparts who received advice. This was similar to a study done in Addis Ababa that concluded that pregnant women who received advice on advantage of early booking were 2 times more likely to book timely compared to pregnant women who did not receive an advice [18]. A study from Uganda also reported similar finding [29]. Therefore, this study suggests that advising pregnant women when to start first antenatal care attendance helps the women to attend antenatal care early. In this study women whose pregnancy was unplanned were 4.5 times more likely to book late for their first ANC as compared to their counterparts. This finding is in line with the study done in south west Ethiopia which stated that unintended pregnancy was 25% and 33% lower to use antenatal care services and receive adequate antenatal care, respectively [10]. Furthermore, studies indicate that unwanted pregnancies strongly associated with late first antenatal care booking or less frequent antenatal care visits, when compared with pregnancy reported as wanted, increased receiving of antenatal care before the sixth month of gestation, respectively [10, 17, 30].

Our study also examined the relationship between household food security status and timely use of antenatal care. Women from food-insecure households were nearly 5 times more likely to start ANC late as compared to women from food-secure households. In food-insecure situations, as food becomes the first priority, expenditures on other goods and services could also be foregone to spare money for buying food. These conditions might be unsuitable for pregnant women to utilize maternal health services like early antenatal care visits. In line with this, other studies indicate that food insecurity has been associated with poor pregnancy outcomes, reduced number of antenatal care visits, decreased self-rated health status, and change of behavior [31–33]. Qualitative studies showed that food-insecure pregnant women worry about having enough food to feed themselves, their unborn children, and their family [34].

This study has some limitations. One of the limitations is the fact that pregnant women who attend antenatal care at private health facilities are not included in the study. Moreover, gestational age was determined based on women's reports of their last menstrual period (LMNP). Ultrasound scan to confirm gestational age was not performed; hence, this may have caused inaccuracies in measurement of gestational age. Moreover, this is a facility based cross-sectional study whose findings are not generalized to a general population. As a cross-sectional study, the associations observed between the explanatory variables and the outcome do not show causal relationship.

## 5. Conclusions

The study showed that more than three-fourth of the pregnant women started ANC in study area. Pregnant women's knowledge of importance of antenatal care for the health of mother and fetus was found to be high. Most of the reasons given by pregnant women who attended antenatal care late were due to perception of appropriate time and shortage of time. This study indicated that low monthly income, women who did not receive advice on when to start antenatal care visits, household food insecurity, and unplanned pregnancy were factors associated with late first antenatal care booking. Based on the findings, it is important to provide continuous health education on importance of early antenatal care visits at health facility.

## Figures and Tables

**Table 1:** Sociodemographic characteristics of pregnant women in Arba Minch Town and Arba Minch District public health facilities, SNNPR, South Ethiopia, March 2014^1^.

Variable (*n* = 409)	*n* (%)
Age, year	
15–24	169 (41.3)
25–34	208 (50.9)
35 and above	32 (7.8)
Ethnicity	
Gamo	311 (76.0)
Welaita	44 (10.8)
Amhara	28 (6.8)
Other^2^	26 (6.4)
Religion	
Orthodox	193 (47.2)
Protestant	192 (46.9)
Other^3^	24 (5.9)
Marital status	
Single	11 (2.7)
Married	384 (93.9)
Other^4^	14 (2.4)
Occupation	
Government employed	48 (11.7)
Self-employed	59 (14.4)
Housewife	293 (71.6)
Other^5^	9 (2.3)
Education status	
No education	117 (28.6)
Primary school (1–8)	134 (32.8)
Secondary school (9-10)	88 (21.5)
Above secondary school	70 (17.1)
Residence	
Urban	175 (42.8)
Rural	234 (57.2)
Household income	
<712.5 Ethiopian birr	118 (28.9)
712.5–1425 Ethiopian birr	107 (26.1)
>1425 Ethiopian birr	184 (45.0)
Family size	
≤4	285 (69.7)
>4	124 (30.3)

^1^All numbers are *n* (%) unless otherwise indicated; ^2^Konso, Oromo, Selte, Gurage, Male, and Tigray; ^3^muslim, catholic, and ahizab; ^4^cohabitation, divorced, and windowed; ^5^student and daily worker.

**Table 2:** Multiple logistic regression models identifying factors associated with timing of first ANC attendance among pregnant women in Arba Minch Town and Arba Minch District public health facilities, SNNPR, South Ethiopia, March 2014.

Variables (*n* = 409)	First ANC attendance		Crude OR (95% CI)	Adjusted OR (95% CI)
	Attended late	Attended early		
Place of residence				
Rural	209	25	2.98 (1.75, 5.08)	1.30 (0.68, 2.48)
Urban^®^	129	46	1	1
Educational status				
No education	108	9	3.24 (1.55, 6.75)	2.1 (0.92, 4.79)
Have education^®^	230	62	1	1
Occupational status				
Government^®^	35	13	1	1
Self-employed	46	13	1.32 (0.54, 3.18)	1.29 (0.48, 3.41)
Housewife	257	45	2.12 (1.04, 4.32)	1.45 (0.62, 3.93)
Monthly income				
≤712.5 ETB	113	5	5.88 (2.24, 15.43)	4.91 (1.72, 14.07)^*∗*^
712.5–1425 ETB	79	28	0.73 (0.42, 1.28)	0.64 (0.34, 1.18)
≥1425 ETB^®^	146	38	1	1
Family size of HH				
Less than five^®^	224	61	1	1
More than five	114	10	3.11 (1.53, 6.28)	1.53 (0.68, 3.43)
Gravida				
Primigravida^®^	118	34	1	1
Multigravida	220	37	1.71 (1.99, 2.87)	0.85 (0.27, 2.67)
Parity				
Parity zero^®^	132	40	1	1
Parity one and above	206	31	2.02 (1.20, 3.38)	1.11 (0.58, 2.07)
Number of children died				
No children died^®^	276	68	1	1
One died and above	62	3	5.09 (1.55, 16.73)	1.41 (0.36, 5.53)
Previous ANC use				
Yes^®^	194	33	1	1
No	144	38	0.65 (0.38, 1.09)	1 (0.30, 3.46)
Advised on when to start (*n* = 280)				
<4 months^®^	102	30	1	1
≥4 months	133	15	2.61 (1.33, 5.10)	3.03 (1.47, 6.24)^*∗*^
Number of ANC visits				
Less than four visits	107	12	2.27 (1.17, 4.41)	1.12 (0.53, 2.43)
Four and above	231	59	1	1
Types of pregnancy				
Planned^®^	208	61	1	1
Unplanned	130	10	3.8 (1.88, 7.71)	4.49 (2.16, 9.35)^*∗*^
HH food status				
Food-secure^®^	296	69	1	1
Food-insecure	42	2	4.9 (1.15, 20.71)	4.66 (1.01, 21.59)^*∗*^
Satisfaction				
High	166	46	1	1
Low	172	25	1.91 (1.12, 3.24)	1.5 (0.83, 2.73)

®, reference category. Selection criteria in binary logistic regression at *p* ≤ 0.25 and ^*∗*^at *p* ≤ 0.05 is considered as statistically significant in multiple logistic regression model. OR: odds ratio.
